# Scoping review of 30 years of suicide prevention in university students around the world: efficacy, effectiveness, and cost-effectiveness

**DOI:** 10.1186/s41155-022-00227-x

**Published:** 2022-07-19

**Authors:** Hareli Fernanda Garcia Cecchin, Sheila Giardini Murta, Etiene Oliveira Silva de Macedo, Rafael Alberto Moore

**Affiliations:** 1 Universidade Federal do Tocantins, Pró-reitoria de Assuntos Estudantis – PROEST, Quadra 109 Norte, Avenida NS-15, Prédio da Reitoria, Plano Diretor Norte, 77001-090 Palmas, Tocantins Brazil; 2 Department of Clinical Psychology, University of Brasília, Brasília, Brazil

**Keywords:** Suicide prevention, Young people, College, Universities, Efficacy, Effectiveness, Scoping review

## Abstract

A scoping review of systematic reviews was carried out to identify evidence of efficacy, effectiveness, and cost-effectiveness of universal and selective suicide prevention programs among university students worldwide. Five databases were reviewed using terms in English, Spanish, and Portuguese. The following were the inclusion criteria: systematic review or meta-analysis or meta-synthesis, suicide prevention in college students, evaluation of the efficacy, effectiveness and/or cost-effectiveness of interventions, and peer-reviewed studies. The quality of reviews was assessed. The field of study features three decades of publication in high-income countries. The strategy used, the components of the program, and the target audience to which they are delivered interfere with efficacy. In the psychoeducation strategy, the experiential and didactic components are more efficacious in the knowledge about suicide. And the motivational enhancement component promotes greater self-efficacy in suicide prevention. Programs that take a multimodal approach are effective in increasing short-term attitudes related to suicide and reducing rates of completed suicide. The gatekeeper strategy delivered to peer counselors is the most effective one in the outcomes, including short-term and long-term knowledge about suicide and its prevention and self-efficacy in suicide prevention. A greater number of evaluated studies of gatekeeper interventions were identified, indicating a trend in this research field. No review addressed the effects on subgroups that were classified based on sex, racial or sexual minorities, and special (indigenous) populations. Only one study addressed cost-effectiveness, pointing out that the psychoeducation and gatekeeper strategies have relevant net benefit rates, but the gatekeeper strategy has a higher cost–benefit ratio compared to the psychoeducation strategy. The findings indicate that psychoeducation and gatekeeper interventions tend to be more efficacious when they combine education and skills training to intervene in suicidal behavior. The components of the intervention and the target audience to which it is delivered influence efficacy. Multimodal interventions evaluate completed suicide outcomes, but require greater implementation efforts, in terms of human and financial resources and more time for the evaluation.

**Supplementary Information:**

The online version contains supplementary material available at 10.1186/s41155-022-00227-x.

## Background

Suicide is the second leading cause of death among young people aged 15 to 29 years worldwide (World Health Organization, 2016). Among university students, suicide is the second leading cause of death in international statistics (Santos et al., 2017). A recent meta-analysis of a sample of countries on several continents showed that college students have a 7.2% lifetime prevalence of suicidal ideation, and suicidal behavior predicts lower performance during university/college years (Mortier et al., 2018). These data indicate the need to implement suicide prevention programs in universities. The literature points to successful initiatives that can guide decisions to implement evidence-based programs, which is the subject of this study.

Universities are a favorable environment for providing preventive programs. This type of institution comprehends a large contingent of young people who are relatively easy to access. On the other hand, young people with suicidal ideation are often reluctant to seek help from traditional mental health services (Perry et al., 2016). Thus, implementing interventions that are focused on modifiable risk factors facilitates optimizing the allocated resources (Harrod et al., 2014). Therefore, the design and wide offer of viable and youth-friendly universal suicide prevention programs represent a relevant target for investment around the world and, in particular, in the context of the  Latin American continent.

A considerable number of studies have been published on suicide prevention, focusing on specific contexts, strategies or populations. Some systematic literature reviews have addressed the issue by evaluating the results of interventions delivered at schools (Balaguru et al., 2013; Katz et al., 2013; Mo et al., 2018) or at schools and universities (Calear et al., 2016; Holmes et al., 2019; Perry et al., 2016). Their findings indicate that interventions improved gatekeepers’ knowledge, attitudes, self-efficacy, skills, and likelihood to intervene. Evidence of achieving improvement in attitudes and gatekeeper behavior was mixed. However, the reviews addressing schools and universities present the findings together, and it is not possible to assess the results only in universities. Furthermore, the results are confined to high-income countries, which limits generalizing the results.

Epidemiological data demonstrate that efforts are important to prevent suicide in low- and middle-income countries. Between 2000 and 2019, suicide rates decreased in all continents, with the exception of the Americas, where there was a 17% increase in the number of occurrences (World Health Organization, 2021). In Brazil, between 2007 and 2017, more than 70% of suicide attempts occurred in people under 40 years old (Ministério da Saúde, 2019). Harrod et al. (2014) conducted extensive research on universal and selective suicide prevention programs at universities, published exclusively in English. The results tracked studies conducted in the USA and Australia, and knowledge related to suicide increased in post-test psychoeducation strategies and gatekeeper, with a reduction in student suicide in a multimodal program. Despite the relevance of the findings of these reviews for the design of suicide prevention programs in young people, context specifics were omitted in the Latin American universities (Harrod et al., 2014). Context is fundamental, both technically and ethically, to design suicide prevention programs. The Latin American context is characterized by deep social inequalities, public policies aimed at reaching historically excluded populations (Afro-descendants, indigenous people and people with disabilities), high truancy rates, difficulty in accessing public health services, among other factors.

Suicide prevention programs for university students use psychoeducation strategies, gatekeeper, screening, restriction of lethal means, or a multimodal approach. The psychoeducation strategy incorporates into school curricula themes related to information about mental health, life skills, suicide prevention, and reducing stigma about mental illness, increasing the probability of a student asking for help when needed (Harrod et al., 2014). The gatekeeper strategy is meant to empower members of the school community to identify and help at-risk students by referring them to health professionals. In general, this strategy has two components: education (increasing knowledge about suicide and its prevention) and training (transmitting skills to intervene in suicidal behavior) (Holmes et al., 2019; Michie et al., 2011; Wolitzky-Taylor et al., 2019). The screening strategy uses assessment tools to identify students who have worrying levels of anxiety, depression, alcohol, and other drug abuse, or some risk of suicide. It is generally combined with the provision of health care for students who need some type of treatment, be it short- or long-term treatment. Reducing access to lethal means can reduce the individual’s opportunity to engage in target behavior (Michie et al., 2011) by restricting access to lethal substances (such as laboratory cyanide), modifying physical structures to prevent falls (Bennett et al., 2015; Schwartz, 2006), among others. Finally, the multimodal approach incorporates two or more of the aforementioned strategies simultaneously, adopting a systemic view and, in general, combining universal, selective, and indicated prevention (Robinson et al., 2018), enhancing the effects of interventions.

However, it is not always clear which interventions are efficacious and which components should be used to design a suicide prevention program for these target audience. Efficacy is the extent to which an intervention does more good than harm when delivered under optimal conditions, and effectiveness is the effect of that intervention when delivered with variations to the implementation team, populations, time, and format (Gottfredson et al., 2015). Cost-effectiveness is measured in terms of how much investment is needed to achieve the observed change in the outcome (Gottfredson et al., 2015) and should be included in evaluations of youth suicide prevention programs (Calear et al., 2016) to assist decision makers.

Therefore, the general objective of this review is to identify the evidence of efficacy, effectiveness, and cost-effectiveness of universal and selective suicide prevention programs for university students. And the specific objectives are as follows: (a) investigate which components of suicide prevention programs are predictors of efficacy, effectiveness, and cost-effectiveness in suicide prevention; (b) identify whether factors of the target population can influence the efficacy and/or effectiveness of the programs; (c) evaluate the quality of systematic literature reviews found; (d) demonstrate the number and origin of suicide prevention programs for university students published worldwide; and (e) investigate whether there are suicide prevention programs with an evaluation of efficacy, effectiveness, and/or cost-effectiveness in Latin American countries. It is expected that these data can support decision-making for researchers, managers, and health professionals interested in designing and offering suicide prevention programs for young university students.

## Method

### Study design

The methodology used was scoping review (Arksey & O'Malley, 2005). This method helps to systematically map the main scientific evidence available in a study area but does not include an assessment of the quality of studies (Colquhoun et al., 2014; Peters et al., 2015; Tricco et al., 2018) so as to contribute to professional practice. The studies were mapped based on interventions located by systematic literature reviews, a kind of umbrella review or accelerated knowledge synthesis (Bennett et al., 2015).

### Search procedures

The articles were located in two stages. In the first stage, searches were performed in electronic databases: BVS (*Biblioteca Virtual em Saúde* - VHL Virtual Health Library), Cochrane Library, ERIC, PubMed, and SciELO in January 2020. The SciELO database was consulted because it is the main digital library in Latin America, as most journals are indexed there. As the objective was to access all available literature, no time and language limits were applied. The review article filter was applied (in databases that had this resource).

The search terms in English, Portuguese, and Spanish were used with the following search strategy: [*suicide OR suicidio AND (prevention OR prevencao OR intervention OR program OR treatment OR therapy OR strategy OR suicide prevention) AND (college OR university OR campi OR school OR campus OR university OR universidade OR universitary OR student OR estudante OR school-based OR university-based OR faculty) AND (eficacia OR eficácia OR efficacy OR efetividade OR effectiveness OR cost-effect)))].* The search strategy was designed and refined by two experienced librarians at the University of Brasília.

In the second stage, searches were performed in the bibliographic reference lists of the articles retrieved in the previous stage, with the objective of maximizing the reach of available published studies. The search took place in the references that had the term “review” and “suicide” in the title. The complete database is available upon request from the first author of this research.

### Selection criteria

The inclusion criteria used were as follows: (a) studies that presented results of systematic review, meta-analysis, or meta-synthesis on interventions for suicide prevention among university students; (b) studies that presented data on the efficacy, effectiveness, and/or cost-effectiveness of interventions; and (c) peer-reviewed studies. The exclusion criteria were as follows: (a) studies that addressed the impact of news about university students’ suicide and (b) studies that addressed indicated prevention, treatment, and intervention in suicidal crisis. These studies were excluded because they focus on major media news, which universities have no control of, and which cannot be targeted by suicide prevention programs in the educational environment. And because in cases of indicated prevention, treatment, and crisis intervention, universities tend to refer to the health network, given the complexity of the case.

In study cases with very specific themes, such as the use of the Internet or work with indigenous populations, the study was only included if the results of suicide prevention programs in university students could be observed separately. Three reviewers completed these tasks independently in duplicate after the training. Disagreements were resolved through consultation with a fourth author.

### Selection process of studies

**Fig. 1:**
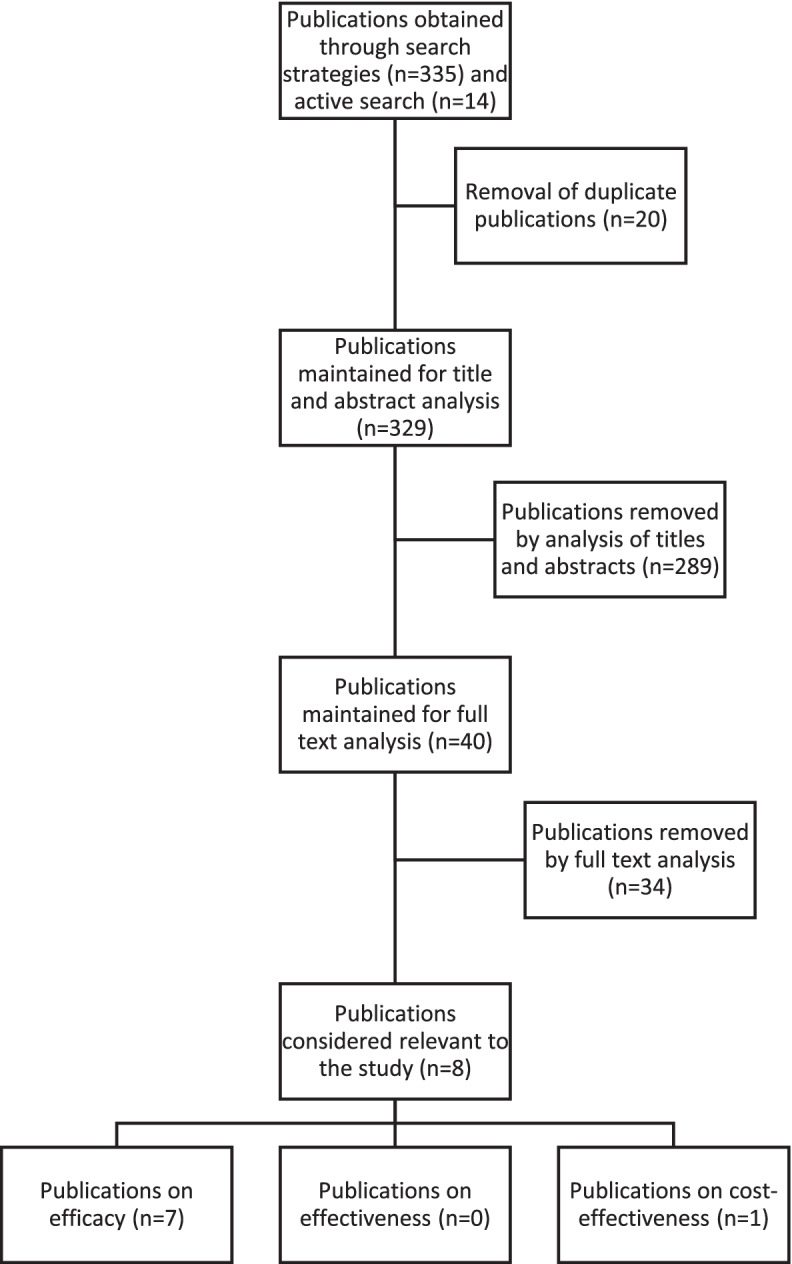
Flowchart of the literature search and inclusion of articles

In the search and screening stages, 335 publications were identified, distributed in the databases as follows: BVS (38), Cochrane Library (26), ERIC (42), PubMed (217), SciELO (12). Fourteen articles are added to this number, located based on the references of the retrieved studies, constituting a sample of 349 publications. After eliminating 20 duplicate studies, a total of 329 references remained. The databases were searched based on their respective start dates until January 2020. After reading the title and abstracts of the articles, and applying the inclusion and exclusion criteria, 289 studies were discarded, and 40 studies were kept for analysis of the full text. After analysis of the full text, literature reviews in which data from university students were indistinguishable from data from other samples were eliminated, totaling 8 studies selected and included in this scoping review, as shown in the flow diagram according to oriented in Peters et al. (2015) (Fig. 1).

### Data collection and analysis

A data mapping form was jointly developed by two reviewers to determine which variables to extract. A reviewer independently mapped the data, discussed it with the group, and continually updated the form in an iterative process. Microsoft Excel software, which allows creating and editing spreadsheets, was used.

The following information was extracted: country and period of publication, quality of systematic literature review, strategy or approach (gatekeeper, psychoeducation and multimodal), type of assessment (efficacy, effectiveness or cost-effectiveness), intervention characteristics, delivery mode (face-to-face or online), characteristics of the target population, outcome measures, and gaps in the studies.

The quality of knowledge synthesis methods was evaluated using the AMSTAR (Assessment of Multiple Systematic Reviews) tool. This tool was created and validated to assess the methodological quality of systematic reviews (Shea et al., 2007). An independent reviewer evaluated the quality of reviews via the AMSTAR protocol.

The literature guided the research team’s decisions. The use of the terms efficacy and effectiveness was analyzed based on the criteria of Gottfredson et al. (2015), by two independent reviewers who mapped and compared the data. Disagreements were discussed with a third reviewer. The Behavior Change Wheel approach was used to define the type of intervention (education and training) (Michie et al., 2011).

In interventions that used the gatekeeper strategy, the extraction of outcome data followed the concepts of Holmes et al. (2019, p.4): (a) knowledge—declarative knowledge and perceived knowledge; (b) self-efficacy—self-efficacy, confidence, and self-perceived competence; (c) attitude—attitude, beliefs, and stigma; (d) behavioral intent—willingness to intervene, preparedness to intervene, probability to intervene, reluctance to intervene, and readiness to intervene; (e) gatekeeper behavior—recognition (of signs of suicide), intervention (asking an individual if they are having suicidal thoughts), and the use of referral pathways (care referrals). It can also be called applying knowledge and implementing skills.

Data were analyzed based on a narrative synthesis. Results were grouped by type of assessment, type of strategy and/or approach, and effects on outcomes. Post-test comparisons of suicide-related outcomes immediately after the intervention were considered short-term. If there was a follow-up evaluation, the results were considered long-term. Evidence was presented in narrative format and in tables.

## Results

The documentary base of this study consisted of 8 literature reviews, published between the years 2008 and 2019, covering a total of 135 interventions described in 131 studies. However, some interventions mentioned in the literature reviews did not address the primary suicide prevention among university students, but were addressed with other audiences or in other institutions. Thus, only 24 interventions were analyzed in this scoping review. Some studies (Abbey et al., 1990; Pasco et al., 2012, Tompkins & Witt, 2009) were mentioned in more than one literature review.

Of the 8 systematic literature reviews, 7 addressed the efficacy of suicide prevention programs for university students and 1 review assessed the cost-effectiveness of interventions. No studies on effectiveness were found. The studies found were published in the USA, Australia, and the UK. The number of published articles increased over the 30-year study period: 1 article was published in the 1980s, 2 in the 1990s, 7 in the 2000s, and 14 in the 2010s. The literature reviews did not track studies published in countries in Latin America, Africa, and Asia.

Regarding the quality, six of the 8 reviews were considered to be high-quality reviews (AMSTAR score ≥7), as detailed in Additional file 1. For more details on this score, see Shea et al. (2007). The main features of the reviews included are summarized in Table 1.

**Table 1:** Characteristics of the included suicide prevention reviews

Revision (author and year)	AMSTAR score	Total studies included	Studies applied at universities	Years surveyed	Terms used by authors	Type of evaluation carried out
Harrod et al. (2014)	11	8	8	NLT to 2011	Effects	Efficacy
Yonemoto et al. (2019)	8	16	1	NLT to 2017	Effectiveness	Efficacy
Zechmeister et al. (2008)	8	14	1	NLT to 2007	Cost-effectiveness	Cost-effectiveness
Kreuze et al. (2017)	7	16	1	NR to 2015	Effectiveness	Efficacy
Kutcher et al. (2017)	7	6	1	NLT to 2017	Effectiveness	Efficacy
Robinson et al. (2018)	7	21	4	NR to 2016	Types of evidence	Efficacy
Witt et al. (2019)	6	39	1	NLT to 2017	Effectiveness	Efficacy
Wolitzky-Taylor et al. (2019)	5	15	15	2000–2018	Effects	Efficacy

*AMSTAR* A tool to assess systematic literature reviews, *NTL* no time limit, *NR* not reported

The term effectiveness was used differently from the literature in three studies. Kutcher et al. (2017), Yonemoto et al. (2019), and Witt et al. (2019) used the term effectiveness, but measured efficacy in different groups separately because they did not assess the degree to which results are generalized in terms of assessing the effects of interventions in different settings and target populations with a statistical analysis of effects in the subgroup and comparison between them, as suggested by Gottfredson et al. (2015).

Only two reviews performed meta-analysis and presented the interventions effect size (Harrod et al., 2014; Wolitzky-Taylor et al., 2019). Therefore, data on the effect size of the studies located could not be detailed and compared. Table 2 shows a summary of the intervention objectives and the main findings. There were also no evaluations of the same program in terms of efficacy, effectiveness, and cost-effectiveness. Due to this, the results on efficacy and cost-effectiveness will be treated separately.

**Table 2:** Main results of systematic reviews on suicide prevention interventions in university students

Authors (publication year)	Study objectives	Main findings
Harrod et al., 2014	To evaluate the effect on suicide and suicide-related outcomes of suicide prevention interventions targeting undergraduate students.	Psychoeducation and gatekeeper strategies increased knowledge related to suicide in the short term. Limited evidence suggested minimal long-term gatekeeper effects on suicide-related awareness. The studies identified did not evaluate the effect of intervention on suicide attempts, threats or suicidal ideation, or help-seeking behavior. Only one study evaluated the effect of the intervention on completed suicides. There is lacking evidence regarding the effects of universities’ institutional policy on suicide prevention. Some gatekeeper interventions involved training peer counselors in on-campus housing, which may have limited applicability to students living off-campus or on non-residential campuses. It is not known whether improved knowledge or attitudes towards suicide persist over time. There is a lack of data to analyze the effects of programs on student subgroups with respect to class, gender, on-campus or off-campus residency, among others. The generalization of results is limited because all studies were applied in high-income countries. There is insufficient evidence to support widespread implementation of the analyzed programs.
Kreuze et al., 2017	Identify how technology-enhanced interventions address the determinants of suicidal behavior.	Technology-enhanced programs have shown efficacy in reducing suicidal ideation and comorbidities. Large-scale research and evaluation initiatives are needed to measure the costs and long-term impact of these interventions on the population.
Kutcher et al., 2017	To analyze the most applied suicide prevention programs in Canada to determine the evidence of effectiveness and safety.	The localized programs (SOS, Yellow Ribbon and SafeTALK) did not show sufficient evidence regarding efficacy or safety. Although the programs are popular in Canada and widely marketed, they do not show evidence related to effectiveness, which may represent a misuse of public resources. Future studies should replace distal outcomes (such as improved social cohesion, self-confidence when discussing suicide, or better knowledge related to suicide) with proximal outcomes (suicide rates or hospital admissions for suicide attempts) to measure the impact of interventions. Research should evaluate what works, what does not, and what causes harm. In addition to evaluating and valuing interventions that deal with the phenomenon globally (better access to clinical care for young people with mental disorders and training of stakeholders [such as teachers and health professionals] to determine risk and intervene appropriately).
Robinson et al., 2018	Synthesize evidence from suicide prevention interventions in schools and universities.	The number of studies carried out in school environments far exceeds those carried out in universities. Overall, 70% of school studies and 50% of university studies showed positive effects on suicidal thoughts and/or behavior. Interventions that used writing showed no reduction in suicidality. New high-quality research must be conducted in university settings, because of the lack of studies in the area. Future research should consider whether the programs have iatrogenic effects.
Witt et al., 2019	Analyze studies that report universal interventions targeting mental health, suicidal ideation and behavior in medical students.	Brief mindfulness-based stress management interventions were effective in reducing levels of anxiety, depression, and stress in medical students in the short term. There should be more studies about the long-term effects on these outcomes, and particularly for suicidal ideation and behavior. Future studies should implement strategies that take into account organizational stressors such as the competitive culture that rewards overwork and permissive environments of intimidation and bullying.
Wolitzky-Taylor et al., 2019	Examine the effects of universal and indicated suicide prevention programs on college students.	Interventions using the gatekeeper strategy had especially large effect on knowledge about suicide and self-efficacy in coping with suicide risk and moderate effect on ability to cope with suicide risk. Online screening and counseling programs have produced promising changes in attitudes but have been limited in their effectiveness with regard to changing help-seeking behavior and linking it to treatment. The authors present 5 suggestions for standardizing the research methodology. And they highlight the importance of evaluating the effects in a multilevel context, such as the number of students with suicidal ideation who were assisted after implementing the gatekeeper training.
Yonemoto et al., 2019	Investigate evidence related to effectiveness of gatekeeper programs in suicide prevention.	The effects of gatekeeper programs are still unclear, even for knowledge, assessments, and self-efficacy after training, although the strongest evidence comes from uncontrolled studies. Only one study evaluated suicidal behavior in the target population as an outcome. Some programs were developed based on theoretical and standardized foundations (such as the QPR program), but others were developed in original contexts or were not clear in their theoretical foundations. In future studies, primary and secondary outcomes should be clearly identified and the referral of people at risk of suicide to appropriate medical resources should be evaluated. The methodology should be standardized so that randomization methods are more clearly described (e.g., generate random sequence and allocation concealment).
Zechmeister et al., 2008	Identify and analyze economic evaluations of mental health promotion and mental disorder prevention interventions.	Only some of the studies reviewed provide strong evidence that preventive interventions are cost-effective. The clearest evidence is in early intervention programs for children and adolescents. There are few studies that analyze the cost-effectiveness of suicide prevention programs. Interventions to promote mental health and prevent mental disorders have a high potential to bring economic benefits to society. However, as the evidence base is scarce, it is difficult to formulate recommendations about prioritizing interventions and transferring results to different contexts.

### Efficacy

#### Aspects related to intervention strategies and their characteristics

Regarding the strategy used, most studies used gatekeeper training, and most of them applied programs formatted and distributed by private companies such as QPR (Question, Persuade, Refer), ALIVE, CampusConnect, and SafeTALK. Efforts to adapt these programs to the local reality or the previous needs of the institution and the target audience were not mentioned.

Most studies were delivered in person. Only a few multimodal programs had online screening and primary care, and a psychoeducation program was delivered online. There were no studies found that used screening as the only type of strategy. One study carried out a cost-effectiveness evaluation comparing the psychoeducation strategy with the gatekeeper one. Given the specifics of each strategy, they will be addressed separately, with outcomes and characteristics.

### Psychoeducation

Psychoeducational interventions had the following short-term results: increased knowledge about suicide, increased knowledge of suicide prevention, and small increase in suicide prevention self-efficacy. Long-term evaluations were not described.

The interventions used at least one of these components: didactic (reading and offering printed material), experiential (modeling and dramatization of suicide prevention situations), and motivational enhancement (use of techniques adapted from motivational interviews to help participants see suicide as a personal relevant issue).

Sensitivity analyses concluded that components mentioned influenced the effect rates on some outcomes (Harrod et al., 2014). The experiential component increased suicide knowledge more than the didactic component. The motivational enhancement component had a small increase in suicide prevention self-efficacy relative to the other two components (Harrod et al., 2014).

Only three psychoeducational studies were found in this study. This may be because 3 of the 8 systematic reviews focused only on gatekeeper programs (Kutcher et al., 2017; Wolitzky-Taylor et al., 2019; Yonemoto et al., 2019). In all interventions, the participants were Psychology undergraduate students.

### Gatekeeper approaches

The gatekeeper strategy had the following short-term results: increased knowledge about suicide, divergent results on knowledge of suicide prevention, divergent results on self-efficacy of suicide prevention, no effect on attitudes towards suicide, and increase in behavioral intent (Harrod et al., 2014; Kutcher et al., 2017; Wolitzky-Taylor et al., 2019; Yonemoto et al., 2019). Follow-up (long-term) assessments occurred up to 6 months after the intervention, identifying the following: increased knowledge of suicide prevention, no effect on self-efficacy in suicide prevention, and no effect on gatekeeper behavior. However, sensitivity analyses concluded that the audience to which the intervention was delivered influenced the effect rates of some outcomes (Harrod et al., 2014), which will be reported below.

In the outcome knowledge about suicide, in all studies there was an increase in the short term (Harrod et al., 2014; Wolitzky-Taylor et al., 2019; Yonemoto et al., 2019). In one study, the intervention was delivered to volunteer and non-volunteer students, and the group of volunteers showed greater effect (Harrod et al., 2014).

In the outcome knowledge of suicide prevention, there were divergent results in the short term, with studies showing an increase and others showing no evidence of effect. The effect varied according to the audience to which the program was delivered: no evidence for the faculty, significant effect for students, and significant effect size in programs delivered to peer counselors (Harrod et al., 2014; Kutcher et al., 2017; Yonemoto et al., 2019). No significant evidence on knowledge of suicide prevention was observed in a program delivered to long-term peer counselors (Harrod et al., 2014).

In the outcome self-efficacy of suicide prevention, divergent results were found in the short term, with significant increases in certain interventions and no effect in others. In the post-test, there was an increase in two programs delivered to peer counselors (Harrod et al., 2014; Wolitzky-Taylor et al., 2019; Yonemoto et al., 2019). In another study, this outcome had a large effect size in seven of the eleven interventions screened, but the authors did not perform a sensitivity analysis to assess the effect difference for each audience (Wolitzky-Taylor et al., 2019). And there was a small and statistically non-significant effect in a program delivered to students, with a small non-significant difference between volunteer students compared to non-volunteer students (Harrod et al., 2014). In the long term, one of the programs delivered to peer counselors showed no evidence of effect (Harrod et al., 2014) and the others did not present this type of assessment.

In the attitudes towards suicide outcome, there was no significant short-term effect either in a program delivered to faculty and staff or in a program delivered to volunteer and non-volunteer students (Harrod et al., 2014). There was no long-term evaluation.

The behavioral intention outcome was assessed in the short term in only two interventions, one delivered to peer counselors and the other to volunteer veterinary students, and an increase was recorded in both (Harrod et al., 2014; Kutcher et al., 2017). However, it is important to highlight that this last study has a high risk of bias, because a pre-test was not applied, nor was there a control group (Kutcher et al., 2017). This program was evaluated in another 5 studies in non-university settings and there was no widespread recommendation by the systematic review authors due to a lack of rigor in the study design (Kutcher et al., 2017). There was no long-term evaluation.

In the gatekeeper behavior outcome, there was no evidence of follow-up effects for two programs delivered to peer counselors (Harrod et al., 2014; Wolitzky-Taylor et al., 2019; Yonemoto et al., 2019). And there was a moderate-to-long term increase in seven of nine studies tracked by Wolitzky-Taylor et al. (2019), but no sensitivity analysis was performed to observe effect differences across different audiences.

The duration of the gatekeeper intervention influenced efficacy, as longer interventions had larger effect sizes (Harrod et al., 2014). This was mainly due to the outcomes of knowledge of suicide prevention and self-efficacy to intervene in someone else’s suicide attempt. All evaluated interventions lasted from 0.5 to 3 h, distributed in one or more meetings over a week.

The gatekeeper strategy was found in seventeen programs presenting a higher number of published interventions and, therefore, a larger database. It also constituted a strategy with a greater number of measured outcomes.

### Multimodal approaches

Programs that used the multimodal approach showed the following long-term results: reduction in completed suicide (Harrod et al., 2014; Robinson et al., 2018), improvement in school performance (Wolitzky-Taylor et al., 2019), moderate size effect on suicidal ideation (Witt et al., 2019), and different effect size on help-seeking behavior and treatment linkage (Kreuze et al., 2017; Wolitzky-Taylor et al., 2019). The studies did not measure the short-term effect.

Programs generally combined screening and psychoeducation or screening and brief treatment, but not exclusively. Due to different arrangements, the studies demonstrate different study designs and measured outcomes. Follow-up assessments ranged from 1 month to 6 years. This range made it difficult to compare the programs, which is why they will be presented individually.

Harrod et al. (2014) found a multimodal program that included restriction to lethal means at the university, gatekeeper intervention with the academic community, and 4 mandatory counseling sessions for students identified with suicidal ideation in the USA. The comparison was carried out with other similar institutions. The suicide rate in the area where the university was located decreased from a rate of 6.91 per 100,000 students enrolled during the 8 years prior to the start of the program to a rate of 3.78 during the first 21 years of the program, which represents a reduction of 45.3%. This reduction occurred against a backdrop of stable suicide rates, both nationally and in a comparison with the other 11 largest institutions in the same location.

Witt et al. (2019) tracked only one intervention that applied screening, gatekeeper training for teachers, psychoeducation for students, and enhancement of individual counseling resources for students in Hawaii. The psychoeducation program had didactic and experiential components. At 1 year of follow-up, there was a moderate reduction in the proportion of participants who reported suicidal ideation (OR 0.07, 95% CI 0.01 to 0.59), with calculations derived from the psychoeducation program (Witt et al., 2019). Faculty members reported having referred more students to university counseling centers, but this increase was not measured numerically.

Kreuze et al. (2017) found a multimodal program that performed online screening, psychoeducation, personalized feedback, and online counseling using motivational interviewing principles. After 2 months, the program increased help-seeking behavior, both for talking to family and friends and to look for a mental health professional. The program also reduced perceived personal and public stigma.

Wolitzky-Taylor et al. (2019) tracked four programs that combined screening (face-to-face or online) and therapeutic counseling. They produced some promising attitudinal changes but were limited in their effectiveness with respect to changing help-seeking behavior and engaging in treatment. In one of these studies, students showed improvement in school performance.

Robinson et al. (2018) found a program that combined screening and psychoeducation, offering a problem-solving workshop for students with depression and mild suicidal ideation. After 1 month, there was a reduction in rates of depressive and suicidal symptoms, but there was no effect on problem-solving ability.

In the short term, in psychoeducation and gatekeeper strategies, there was consistent evidence of positive changes in knowledge about suicide and knowledge of suicide prevention, except for one program delivered to faculty members and staff. Inconclusive findings were identified in the self-efficacy of suicide prevention. An absence of change was found in attitudes towards suicide.

In the long-term, in gatekeeper and multimodal strategies, consistent evidence of positive changes was found in knowledge of suicide prevention, help-seeking behavior, stigma reduction, increased treatment linkage, and reduced completed suicide. Inconclusive findings were identified in the reduction of depressive symptoms. Absence of change was identified in problem solving skills. Tables 3 and 4 summarize the main results by strategy.

**Table 3:** Summary of short-term results by strategy

Strategy	Tracked by	Primary study	Components	Audience	Outcomes				
					Knowledge of suicide	Knowledge of suicide prevention	Self-efficacy in suicide prevention	Attitudes towards suicide	Behavioral intent
Psychoeducation	Harrod et al., 2014	Abbey 1989		Students	Increase	Increase	Small and non-significant increase		
		Abbey, 1989	Experiential and didactic		Increased more than the experiential				
		Holdwick, 2000	Motivational enhancement				Effect size a little larger in relation to the didactic and experiential components		
Gatekeeper	Harrod et al., 2014	Drabek, 1991	Experiential and didactic	Faculty and staff	Increase	No evidence of effect		No evidence of effect	
		Shipley, 2003		Students (volunteers and non-volunteers)	Increase in general	Increase	Small non-statistically significant effect	No evidence of effect	
				Volunteer student	Greater increase related to control	Greater increase in relation to control and non-voluntary	Small non-statistically significant difference between v and nv compared with students who did not receive training	Slight difference, not statistically significant between v and nv compared to control	
	Harrod et al., 2014	Pasco et al., 2012	Experiential and didactic	Peer counselors		Moderate increase	Increase		
	Harrod et al., 2014	Tompkins & Witt, 2009	Does not inform	Peer counselors					
	Yonemoto et al., 2019						Increase		Increase
	Kutcher et al., 2017	Mellanby et al., 2010		Students					Moderate increase
	Wolitzky-Taylor et al., 2019	All			Increase		Increase

**Table 4:** Synthesis of long-term results by strategy

	Tracked by		Components	Audience	Knowledge of suicide prevention	Self-efficacy in suicide prevention	Gatekeeper behavior	Help-seeking behavior (2 months)	Personal stigma and public stigma	Treatment linkage (2 months)	Problem solving skills (1 month)	Rates of depressive and suicidal symptoms (1 month)	Completed suicide (6 years)
Gatekeeper	Harrod et al., 2014	Tompkins & Witt, 2009	Does not inform	Peer counselors	Long-term effect	There was no evidence	No evidence of effect						
	Yonemoto et al., 2019						No effects						
Multimodal	Harrod et al., 2014 e	Joffe, 2008	Reduction of means, gatekeeper, and brief intervention										decrease
	Robinson et al., 2018												
	Kreuze et al., 2017	King et al., 2015	Screening, feedback, and online counseling	Students				Increase	Decrease	Increase			
	Robinson et al., 2018	Fitzpatrick et al. (2005)	Screening and psychoeducation	Students							No effect	Decrease	
	Robinson et al., 2018	Kovac and Range (2002)	Screening and therapeutic writing					Statistically insignificant increase				There was no effect	
	Witt et al., 2019	Thompson et al. (2010)	Psychoeducation, gatekeeper, screening, improvement of counseling services									Decrease (after 1 year)	

### Aspects of the target population

It was not possible to verify whether the characteristics of the target population, such as age, gender, marital status, social class, on-campus or off-campus residency, limit or facilitate efficacy, since the lack of data prevented the analysis from being carried out for relevant subgroups to examine the modification of intervention effects (Harrod et al., 2014). Some programs were carried out in specific undergraduate courses, such as Medicine, Veterinary Medicine, and Psychology. However, due to the low number of interventions with this type of publics, it was not possible to make comparisons between them.

No systematic literature reviews were found that addressed the efficacy of suicide prevention programs in college students that took gender into account. However, in the 24 studies tracked, the participation of women was greater than that of men, ranging from 51 to 82%, in the sample composition. An analysis of the recruitment and selection of participants did not demonstrate any strategy that could influence a greater participation of female students, as information about the intervention was offered the same way to men and women.

In some interventions, it was observed that more motivated individuals result in higher levels of efficacy. In one intervention, the voluntary participation in a QPR workshop (determined by self-reported motivation to participate at the beginning) was associated to better outcomes compared to involuntary participation (Harrod et al., 2014).

### Cost-effectiveness

#### Aspects related to intervention strategies and their characteristics

A study examined the potential impact of offering two prevention programs, one focused on general suicide education (psychoeducation) and the other on peer support (gatekeeper), for university students (Sari et al., 2008). The study found that the evaluated program that used psychoeducation had a cost–benefit ratio of US$ 2.03, effect rate of 57%, and net benefits of US$ 112 million. The program that used the gatekeeper strategy had a cost–benefit ratio of US$3.71, an effect rate of 60%, and net benefits of US$109 million, as shown in Table 5. The study did not evaluate whether certain characteristics of the intervention (format, sample size, duration) are more cost-effective.

**Table 5:** Evaluation of the economic impact of suicide prevention programs

	Benefit–cost ratio	effect rate	Implied net benefits (US$)
**Psychoeducation**	2.03	57%	112 million
**Screening**	3.71	60%	109 million

Source: prepared based on data from Sari et al. (2008)

Based on estimated effect rates for general education (psychoeducation) and peer support (gatekeeper) programs to prevent suicide (57% and 60%, respectively), the authors concluded that the implementation of both programs in all Florida universities would result in net benefits of US$ 21 million for the general education program (psychoeducation) and US$ 32 million for the peer support program (gatekeeper). This demonstrates that the implementation of these programs provides net positive benefits to society. The study did not calculate additional costs related to the support that family and friends may need after the loss of a loved one. These may involve the increased use of mental health resources and the need to take time from work or school, implying in costs for public policies and a decrease in productivity and/or school performance.

#### Aspects of the target population

The study did not present the characteristics of the target population, such as gender proportion in the groups, marital status of the participants, social class, and the presence or absence of ethnic minorities. The age of the target audience for the interventions was between 18 and 24 years old.

## Discussion

The findings of this scoping review indicate that components and participants interventions impact its efficacy. Psychoeducation and gatekeeper interventions tend to be more efficacious when they combine education and training to intervene in suicidal behavior. Members of the university community (such as faculty members and student leaders or volunteers) are important institutional resources that should be considered. However, the results are not sustainable in the long term and therefore actions must be implemented to encourage the maintenance of the outcomes. These strategies demonstrate good results in terms of cost-benefit, but the results cannot be generalized, since only one study was found. Multimodal interventions, on the other hand, did not evaluate the same outcomes as psychoeducation and gatekeeper interventions, making it impossible to compare these strategies.

Gatekeeper training proved to be the strategy with the highest number of published studies, a finding that coincides with another literature review that covered programs in schools and universities (Breet et al., 2021). However, this strategy proved to be efficacious in increasing awareness about suicide and its prevention, but with no effect on informing skills to intervene in suicidal behavior. It may be easier to alter the subjective experience (feeling more confident in approaching suicide) than the objective aspect (how skillfully the individual approaches suicide), which is why it is important to assess how such interventions affect actual performance change (Wolitzky-Taylor et al., 2019). To translate the increase in awareness into supportive behaviors and referrals to health services, it is necessary to understand the behavior and its determinants (Peters, 2014) or fundamental components (Michie et al., 2011), in order to identify levers for change, using appropriate theories and methods. Furthermore, as this strategy is meant to increase help-seeking behavior, it is important that affordable and effective mental health services are available to students (Breet et al., 2021).

Another point that deserves attention is the target audience of the gatekeeper intervention, as better results were found in programs delivered to peer counselors and students who volunteer to participate. Training people who volunteer to participate can be used to select individuals with greater interest in the topic and a greater sense of empathy, enhancing the effects of the intervention and taking advantage of community resources (Holmes et al., 2019). Peer counselors, being veteran fellow students and playing a leadership role, may feel more comfortable intervening in suicide risk situations. On the other hand, it is necessary to be careful not to generate a feeling of responsibility and guilt in young people who face the situation of trying to prevent the suicide of a colleague (Pistone et al., 2019). However, in the long term, the results of the gatekeeper programs were not maintained, which coincides with the finding of another review on the topic (Mo et al., 2018). As for the greater participation of female individuals in the studies, these results are similar to the findings of other international studies (Hamilton & Klimes-Dougan, 2015; Millan & Arruda, 2008), in which women participate more in screening and they value training more gatekeeper and have greater help-seeking behavior.

Psychoeducation and gatekeeper strategies did not measure the effect on intermediate outcomes (increased mental health help seeking) and final outcomes (reduction of completed suicide). Similar results were found in another literature review (Breet et al., 2021; Eisenberg et al., 2012). Seeking help and connected to the treatment could be outcomes evaluated in all types of strategies, allowing to compare them.

Due to its characteristics, multimodal approach studies did not evaluate proximal outcomes such as education and training to intervene in suicidal behavior. In terms of outcomes, available evidence suggests that this is the best bet, albeit the costliest. However, its implementation requires a large amount of human and financial resources and may not be the most suitable in resource-poor contexts. In this case, it would be more appropriate to focus on community competencies and resources since the beginning of the program planning, establishing a partnership with community members and relying on them in all phases of the program (Eldredge et al., 2016; Wallerstein et al., 2017).

It was not possible to verify the efficacy of the screening and restriction strategies, since in all studies they are associated with other initiatives, especially brief intervention (therapeutic counseling). The multimodal interventions presented a challenge in terms of evaluation, in terms of measuring the effects of each strategy, as they are applied jointly and over the same time frame. These interventions can have cascading effects that increase when they reach the next level (van der Feltz-Cornelis et al., 2011). Better recognition of depression (awareness) leads to treatment, which in turn leads to decreased symptoms and a lower incidence of suicidal ideation. More individuals at risk of suicide located and referred for help may require improved health services.

The components of the intervention show interlocutions between knowledge. The results show interaction between universal, selective, and indicated prevention, as motivational interviewing principles used in the treatment (psychotherapy) were implemented in the psychoeducation and multimodal programs. The personalized feedback implemented in a multimodal program delivered online stands out. This reiterates findings about customized interventions being more advantageous than generalist ones, as they provide more identification, attention, and retention (Murta & Santos, 2015).

### Literature gaps

There were some gaps identified in the literature. Most localized reviews used keywords exclusively in English and searched databases linked to high-income countries, which may have prevented localizing the studies published in low- and middle-income countries. This result is similar to another systematic review (Breet et al., 2021). The studies are cautious about generalizing the results, as most studies were conducted in the USA or countries on the European and Australian continents, that is, high-income countries. A publication bias and/or overestimation of the effect sizes of interventions may be occurring in reviews due to language (Egger et al., 1997; Shea et al., 2007). All reviews located in this study used only English search terms, 4 reviews used articles in English or articles published in the USA as inclusion criteria, and only 2 of the 8 reviews located did not restrict the search by language (Harrod et al., 2014; Yonemoto et al., 2019). It is possible that suicide prevention studies were carried out in low- or middle-income countries but were not screened by systematic literature reviews due to a preference for studies published in English. None of the systematic literature reviews indicated the wide dissemination of any program.

In addition to the influence of the language of publication, the data must be observed, bearing in mind the sociocultural and political contexts that can impact suicide prevention initiatives. Silverman et al. (2020) suggest 10 initiatives to improve suicide prevention in the Americas: improve the identification, diagnosis, and rehabilitation of people involved in substance abuse; invest in the training of general practitioners for early identification, intervention, and treatment for depression and suicide attempts; train community members (gatekeepers) to contribute to suicide risk assessment; establish local crisis hotlines; expand resources for research and evaluation; establish intersectoral collaborations, especially between governments and non-governmental organizations (NGOs); invest in public education; offer more attention to services aimed at child/family relationships; reduce access to lethal means, especially in relation to agrochemicals and firearms; and establish national plans with well-defined action strategies. These proposals are in line with the research data by Machado et al. (2014) who point out 7 obstacles to suicide prevention in Brazil: taboo in relation to suicide, lack of public awareness hotlines and help channels, underreporting of cases, failures in care that require training health professionals, easier access to lethal methods, increase in chemical substance abuse, and lack of adequate and more efficient prevention plans that articulate local and national actions.

There was no discussion of cultural differences in any study. The included studies provided little or no information about their samples in terms of sociocultural characteristics such as gender, race or ethnicity. Primary prevention interventions targeting the general population may not be culturally relevant to minority students, reducing their efficacy in these subgroups, and increasing existing health disparities (Harrod et al., 2014). Studies point to a gap in culturally adapted suicide prevention programs for indigenous people, with no studies being found in South America (Nasir et al., 2016). Worldviews, values, and cultural beliefs of those who receive and those who offer an intervention may be different and create opportunities for these actors to interact and influence one another, and in co-creation experiences, it increases the effectiveness of interventions (Davis et al., 2018). Since suicide is intrinsically affected by sociocultural factors, there is no sure indication for generalizing the data to other contexts.

As for the quality of literature reviews, the criteria established by AMSTAR that were not observed in most interventions were gray literature search, list of studies (included and excluded), and tests to assess homogeneity of the combined studies. In addition, the reviews did not seek to evaluate other aspects that may have contributed to the effectiveness of the interventions, such as the theoretical approach used and consulting the stakeholders. Some reviews used the term effectiveness in situations where the term efficaciousness would be more appropriate.

The effects of interventions have not been measured over the long term and it is not known whether they hold. It was not possible to evaluate the effect of psychoeducation and gatekeeper interventions on suicide attempts, threats, or ideation or help-seeking behavior. Few studies have evaluated the effect of using technologies in online screening and counseling. Studies have not reported the capacity of the health system in the territory to meet the mental health demands of university students. Only two meta-analysis studies were found, and only one of them reported the intervention effect size.

There is a lack of diversity in the populations of origin, as only undergraduate Psychology students participated in psychoeducation programs, which can lead to outcome bias, as these students may have been more avid, trained or attentive to intervention, leading to more positive effects on outcomes related to suicide prevention (Harrod et al., 2014).

Only one study was found regarding the cost-effectiveness of suicide prevention programs for college students. One of the likely reasons is the lack of a specific standard for calculating costs, although there are efforts being mobilized to create guidelines in this area (Gottfredson et al., 2015). As of 2015, the intervention cost has been requested in efficacy and effectiveness requirements, but many studies cited in this scoping review were carried out before this date and did not adapt to the changes. Systematic literature reviews found few economic evaluations of suicide prevention and intervention programs overall (Madsen et al., 2018), and no economic evaluation of programs targeting 0- to 24-year-old individuals (Bennett et al., 2015), or in interventions that make use of online platforms and cell phone applications (Franco-Martín et al., 2018), nor in suicide postvention programs (Szumilas & Kutcher, 2011), pointing out a gap in scientific production in this area.

### Implications for research and practice

The results of this study have consequences for research and practice in seven observations (see Table 6 for a summary of proposed suggestions). Replication of these interventions in low- and middle-income countries should be carried out cautiously. The context must be observed and changes may be necessary, which justifies the use of outlines that value or consider the needs of the university community. It is suggested that managers, researchers, and health professionals observe the didactic components and motivational improvement in psychoeducational interventions; the focus on student leaders and volunteers in gatekeeper interventions; and reaching the male audience, as women tend to more easily adhere to suicide prevention programs, but higher suicide rates are found among men.

**Table 6:** Research priorities to promote evidence-based suicide prevention practices in universities

• Consider the context of low- and middle-income countries • Standardize the research methodology • Include initiatives to retain the effects of the gatekeeper strategy • Combine didactic, experiential and motivational enhancement components in psychoeducation programs • Seek male audience engagement • Include a more comprehensive economic assessment	

The eight systematic literature reviews included in this study suggest the need to standardize the research methodology in this area. Authors point out the need for greater methodological rigor in studies seeking to (a) use specific designs such as randomized clinical trials and quasi-experimental designs; (b) use psychometric tools that are consistent across studies to assess core domains; (c) publish complete descriptive statistics of the studies, such as *t* values and sample sizes; (d) evaluate outcomes at specific times, that is, immediately before starting the intervention, immediately after completion of the intervention, and at a follow-up time to determine how well the changes are maintained; and (e) consistently report key variables of interest that could theoretically impact outcomes (Wolitzky-Taylor et al., 2019).

Since the programs that used the gatekeeper strategy did not maintain long-term results, future research should assess this care in order to maintain the effects. These are as follows: offer support networks and the possibility of connecting with other gatekeepers; customize the training considering the level of knowledge of each subpopulation (lay public, health professionals, etc.); and use technologies to provide gatekeepers with up-to-date information on local health services, reminders, ongoing feedback, follow-up materials, and summaries of the intervention (Holmes et al., 2019; Shtivelband et al., 2015). In addition to increasing the number of conscientious people and having a support group for gatekeepers, future research should provide certification as encouragement and perform replacement interventions so that participants have the opportunity to practice their skills (Holmes et al., 2019; Nasir et al., 2016; Shtivelband et al., 2015). Among the intervention components, it is important to address sociocultural aspects of suicide with the objective of changing attitudes, since the increase in knowledge is not enough to increase gatekeeper behavior (Holmes et al., 2019). In the selection of participants, the authors suggest not selecting people with a recent suicidal history to participate in gatekeeper training as supporters (Nasir et al., 2016).

Psychoeducation programs should combine didactic, experiential, and motivational enhancement components to increase their efficacy. In addition to providing education on suicide prevention and promoting useful attitudes, these programs could include knowledge about mental health, mental health disorders, and their treatments and expand the life skills of young people (teaching how to deal with stress, improve communication, and solve problems) (Grosselli et al., 2021). As a suggestion for objectives and content, these authors also mention “fostering the search for help (attitudes, behaviors); improve peer support for suicidal youth; informing about issues related to suicide (e.g., bullying, risk behavior); reduce stigma in relation to mental health disorders and seeking help, and reduce risk factors for suicide attempts (hopelessness, social isolation)” (p.4).

Due to the greater participation of female individuals in the studies, male engagement should be the target of efforts in the design of new suicide prevention programs, especially in terms of increasing the search for professional help (Sagar-Ouriaghli et al., 2020). In multimodal interventions, it may not be productive to offer psychoeducation to young people in emotional distress, as in the study by Fitzpatrick et al. (2005), students with depression and mild suicidal ideation were unable to improve their problem-solving skills. When people are distressed, their cognitive resources are depleted and they may have limited energy to learn (Davis et al., 2018).

For future research, it is suggested to include a more comprehensive economic assessment. Future studies can be designed to assess the iatrogenic effects of interventions and measure the long-term effects of the psychoeducation strategy, the cost-benefit, and effectiveness of interventions in low- and middle-income countries. Additionally, it would be beneficial to map the effects of programs on specific subgroups (observing gender, ethnicity, sexual orientation, course period, etc.), populations from resource-poor contexts, and/or traditional populations (such as indigenous and quilombolas).

The main limitation of this study is the restriction in studies covered by systematic literature reviews. We are aware that existing systematic reviews and meta-analyses may be subject to misinterpretations of primary studies, omission of important primary studies, and inclusion of low-quality primary studies by review authors. Our objective is to alleviate this bias by evaluating the quality of systematic literature reviews.

## Conclusions

The findings of this scoping review indicate a lack of studies on the effectiveness and vast production on the efficacy of suicide prevention programs for university students. Evidence on cost-effectiveness is still limited to a just one study with restricted scope for generalizability. However, heterogeneous efficacy results have been observed depending on the strategy used, to which public it is delivered, the analyzed outcome, and the time to measure the results. Current approaches to suicide prevention in the university setting emphasize knowledge and attitudes about mental illness, the development of skills to provide help, and treatment options. However, the basis of evidence for these approaches needs to be significantly strengthened. Furthermore, new approaches must be explored because individual-focused issues such as knowledge and attitudes may not be the main barriers for many students who do not seek help. Ecological and systemic approaches could help to focus on other factors that go beyond the personal and interpersonal dimension, but that consider the influences of the organization, the community, and society in which the individual is inserted, and the social and constructed environment, such as availability and quality of services offered. The context must be observed, especially in low- and middle-income countries, since in these locations there may be less access to health services by some sections of the population.

## Supplementary Information

**Additional file 1: Table 1.** Evaluation of systematic literature reviews according to the AMSTAR criteria.

**Additional file 2.**

## Data Availability

The datasets used and/or analyzed during the current study are available from the corresponding author on reasonable request.

## Abbreviations

AMSTAR: Assessment of Multiple Systematic Reviews
NR: Not reported
NTL: No time limit
QPR: Question, Persuade, Refer Program
