# Supplementary material for: Scoping review of 30 years of suicide prevention in university students around the world: efficacy, effectiveness, and cost-effectiveness
Source: Psicol Reflex Crit. 2022 Jul 19;35:22. doi: 10.1186/s41155-022-00227-x (PMC9294115; doi:10.1186/s41155-022-00227-x)
Supplement: Supplementary file 1 — Additional file 1: Table 1. Evaluation of systematic literature reviews according to the AMSTAR criteria. [file 41155_2022_227_MOESM1_ESM.docx]

**Supplementary Information**

**Table 1** – Evaluation of systematic literature reviews according to the AMSTAR criteria

|  | Harrod et al. (2014) | Kreuze et al. (2017) | Kutcher et al. (2017) | Robinson et al (2018) | Witt et al. (2019) | Wolitzky-Taylor et al. (2019) | Yonemoto et al., 2019 | Zechmeister et al., 2008 |
| --- | --- | --- | --- | --- | --- | --- | --- | --- |
| **1.** **Was an** ***'a priori*' project provided?** The search and inclusion criteria must be established before the review is carried out. | 1 | 1 | 1 | 1 | 1 | 1 | 1 | 1 |
| **2. Was there duplicate study selection and** **data extraction?** There must be at least two independent data extractors, as well as a consensus procedure for disagreements. | 1 | 0 | 1 | 1 | 1 | 0 | 1 | 0 |
| **3. Was a comprehensive literature search carried out?** At least two electronic sources must be searched. The report should include years and databases used (e.g. Central, EMBASE and MEDLINE). Keywords and/or MESH terms should be indicated and, when possible, the search strategy should be provided. All research should be complemented by consulting current content, reviews, textbooks, specialized records or experts in the specific field of study, and reviewing references in the studies found. | 1 | 1 | 1 | 1 | 1 | 0 | 1 | 1 |
| **4.** **Was publication status (i.e., gray literature) used as an inclusion criterion?** Authors must state that they looked for reports regardless of the type of publication. Authors must state whether or not they excluded any reports (from the systematic review) based on their publication status, language, etc. | 1 | 0 | 1 | 0 | 0 | 0 | 0 | 1 |
| **5.** **Was a list of studies (included and excluded) provided?** A list of included and excluded studies must be provided. | 1 | 0 | 0 | 0 | 0 | 0 | 0 | 1 |
| **6.** **Were the characteristics of the included studies provided?** In a combined form, as a Table, data from the original studies on participants, interventions and outcomes should be provided. Characteristic ranges in all analyzed studies should be reported, for example, age, race, sex, relevant socioeconomic data, disease status, duration, severity, or other diseases. | 1 | 1 | 1 | 1 | 0 | 0 | 1 | 1 |
| **7.** **Was the scientific quality of the included studies evaluated and documented?** *'A priori*' evaluation methods should be provided (e.g., for effectiveness studies if the author chooses to include only randomized, double-blind, placebo-controlled, or allocation concealment studies as inclusion criteria); for other types of studies, alternative items will be relevant. | 1 | 1 | 0 | 1 | 1 | 1 | 1 | 1 |
| **8.** **Was the scientific quality of the included studies properly used to formulate conclusions?** The results of methodological rigor and scientific quality should be considered in the review's analysis and conclusions, and explicitly stated in the formulation of recommendations. | 1 | 1 | 0 | 1 | 0 | 1 | 1 | 1 |
| **9.** **Were the methods used to combine the findings of the studies appropriate?** For combined results, a test should be done to ensure that the studies were combined to evaluate their homogeneity (i.e., chi-square test for homogeneity, I2). If there is heterogeneity, a random effects model should be used and/or the clinical suitability of the combination should be taken into account (that is, is it wise to combine?). | 1 | 1 | 0 | 0 | 0 | 0 | 0 | 0 |
| **10.** **Was the likelihood of publication bias assessed?** An assessment of publication bias should include a combination of graphical support (e.g., funnel plot, other available tests) and/or statistical tests (e.g., Egger regression test). | 1 | 0 | 1 | 0 | 1 | 1 | 1 | 0 |
| **11.** **Was the conflict of interest declared?** Potential support sources should be clearly documented in both the systematic review and the included studies. | 1 | 1 | 1 | 1 | 1 | 1 | 1 | 1 |
| Total score according to the criteria | 11 | 7 | 7 | 7 | 6 | 5 | 8 | 8 |
